# n-Butylamine for Improving the Efficiency of Untargeted Mass Spectrometry Analysis of Plasma Metabolite Composition

**DOI:** 10.3390/ijms20235957

**Published:** 2019-11-27

**Authors:** Dmitry L. Maslov, Oxana P. Trifonova, Elena E. Balashova, Petr G. Lokhov

**Affiliations:** Institute of Biomedical Chemistry, 10 Building 8, Pogodinskaya Street, 119121 Moscow, Russia; oxana.trifonova@gmail.com (O.P.T.); balashlen@mail.ru (E.E.B.); lokhovpg@rambler.ru (P.G.L.)

**Keywords:** metabolome profiling, alkylamines, decontamination procedure

## Abstract

A comparative study of the impact of n-butylamine and traditionally used additives (ammonium hydroxide and formic acid) on the efficiency of the electrospray ionization (ESI) process for the enhancement of metabolite coverage was performed by direct injection mass spectrometry (MS) analysis in negative mode. Evaluation of obtained MS data showed that n-butylamine is one of the most effective additives for the analysis of metabolite composition in ESI in negative ion mode (ESI(−)) The limitations of the use of n-butylamine and other alkylamines in the analysis of metabolic composition and a decontamination procedure that can reduce MS device contamination after their application are discussed. The proposed procedure allows the performance of high-sensitivity analysis of low-molecular-weight compounds on the same MS device in both polarities.

## 1. Introduction

The metabolome, as a collection of low-weight substances (<1000 Da) that are the result of complex interactions of gene and protein expressions, has an influence on various factors (including environment, lifestyle, diet, gut microbiome, drug exposure, etc.) [1]. Electrospray ionization mass spectrometry (ESI-MS) is one of the most widely employed methods for untargeted metabolomic analysis (metabolomic profiling) [2,3]. An analysis of overall metabolomic composition and its alterations in response to various physiological or pathophysiological factors, therapeutic procedures, and so forth, provides a comprehensive overview of an organism’s actual physiological state, which enables revealing the possible mechanisms of disease pathogenesis, potential biomarkers for improving disease diagnosis and prognostication, and so forth [4]. However, there are many factors that limit the broad coverage of metabolite composition in a sample. The wide concentration range of metabolites presented in biological samples, the diversity of physical and chemical properties of the metabolites related to various classes (amino acids, lipids, and carbohydrates), and so forth, prevent the correct detection of numerous sample metabolites by one analytical approach [5,6,7]. The application of various additives to enhance the ESI ionization efficiency of a wide range of metabolites is one of the routine laboratory methods for improving coverage [7,8]. The inclusion of additives in the spray solvent is a simple, fast, and easily reproducible method that enables bypassing the problem of nondetectability of some analytes. Weak organic acids (formic and acetic) are the most common additives for positive ESI mode (ESI(+)). Several salts, which can form [M + Na]^+^ and [M + NH_4_]^+^ analyte adducts, are sometimes applied in experiments as well [9].

In contrast, there is no consensus on the selection of the optimal additives for negative ESI mode (ESI(−)) [8]. The lack of full understanding of the ESI(−) process is a reason for this paradox. Ammonium salts (hydroxide, formate, and acetate) are traditionally used in ESI(−) analysis [10,11]. Among these, a wide range of substances (piperidine, organic acids, etc.) that can significantly enhance ESI ionization efficiency in negative ionization mode have been described [11,12,13,14,15]. The substances are characterized by diverse chemical properties (from organic acids to bases), and often, they demonstrate higher effectiveness compared with ammonium salts. However, most of the investigations of the efficiency of these additives were carried out using targeted analytes [14,15]. Thus, the search for new effective additives for untargeted metabolomic analysis continues.

Secondary and tertiary alkylamines are commonly used as additives in the analysis of different classes of compounds (nucleosides, proteins, phospholipids, nucleic acids, etc.) in ESI(−) [16,17,18,19,20,21]. Unfortunately, there is no information about the application of alkylamines in untargeted metabolomic analysis. We suggest that alkylamines can also increase the MS signal intensity of metabolites in ESI(−). However, the tendency of alkylamines to adhere to the surfaces of an MS device (ion source surfaces, vacuum manifold and parts therein, etc.) and their high proton affinity lead to the suppression of analyte ionization and a reduction in sensitivity for ESI(+). This limits the use of secondary and tertiary alkylamines in the study of low-mass compounds on the same MS device in both polarities [22,23]. We hypothesized that primary alkylamines can increase the efficiency of ionization of metabolites in ESI(−) and, at the same time, unlike secondary and tertiary amines, not lead to persistent MS contamination.

The main goal of our study was the comparative analysis of the ionization efficiency of a primary alkylamine (n-butylamine) and traditionally used additives (ammonium hydroxide (base) and formic acid (weak acid)) to investigate the metabolomic composition in ESI(−).

## 2. Results

A comparative analysis of metabolite profiles of deproteinized pooled human plasma samples using the selected additives was performed by the direct injection mass spectrometry (DIMS) method. Despite the limitations of the approach (ion suppression, etc.), many authors have applied the DIMS procedure for untargeted analysis of complex mixtures [24,25,26]. High throughput, reproducibility, and minimized metabolite losses are the main advantages of this approach [27]. To reduce contamination of the MS device, a concentration of additives was chosen from the range of minimum effective concentrations, which enabled increasing the ionization efficiency [12,28].

The evaluation of the effectiveness of the chosen additives for metabolic profiling was based on a comparison of the total number of detected mass peaks and intensities of randomly selected mass peaks. A preliminary filtration allowed for removing the background peaks (not plasma related). Blank samples (samples containing the same reagents in the same concentration without plasma) were used for this purpose. The blank samples were analyzed by the same direct injection method, and all detected mass peaks (peaks related to plasticizers, additives, components of solvent, etc.) were noted and further subtracted from the list of mass peaks. Only peaks that were detected in each sample of the compared group were admitted for analysis.

**Figure 1:**
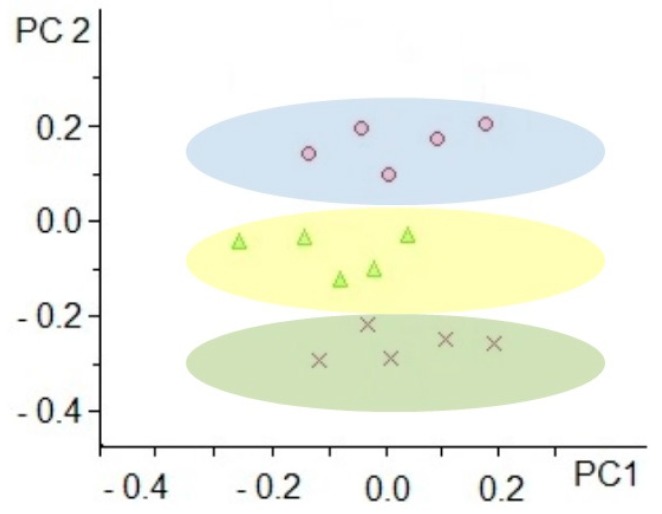
Principal component analysis (PCA) score plot. The score plot was created using the first two principal components which had a maximal explanation of the variances between the analyzed groups (more than 75% of the total variance). The PCA score plot demonstrates the compact clustering of samples according to assigned group membership (samples where formic acid was used as an additive (×), samples where ammonium hydroxide was used as an additive (Δ), and samples where n-butylamine was used as an additive (O)).

Principal component analysis (PCA) of MS data demonstrated the formation of compact clusters in accordance with the sample groups where one of the selected additives was used (Figure 1). The first two components (PC1 vs. PC2), which had a maximal explanation of the variances between the analyzed groups (more than 75% of the total variance), were taken for the creation of the demonstrated PCA model. The high reproducibility of the results of metabolomic profiling between samples in each compared group was the reason for the compact clustering. At the same time, obvious separation between the three group samples was observed.

**Figure 2:**
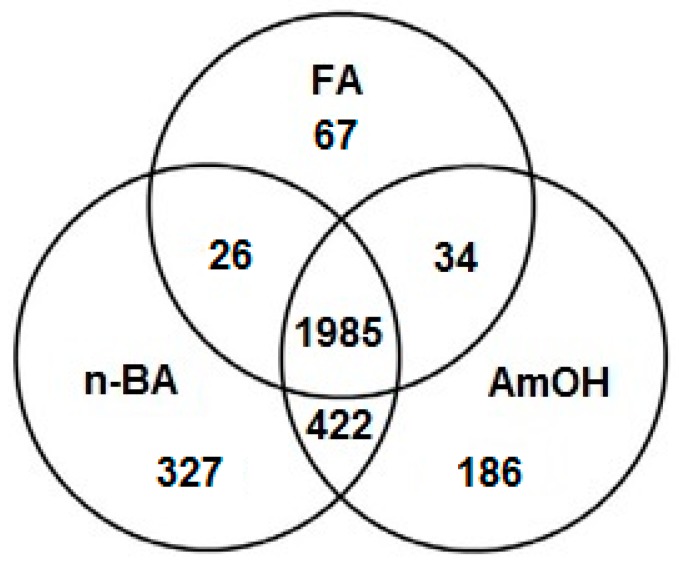
Diagram of mass peaks detected in samples. The Venn diagram presents the overlapped and unique low-molecular-mass peaks detected by direct mass spectrometry profiling in negative mode in blood plasma samples where the studied additives were used. Only peaks that were detected in all samples of each group were admitted to the analysis. Abbreviations: FA—a group of samples where formic acid was used as an additive; n-BA—a group of samples where n-butylamine was used as an additive; AmOH—a group of samples where ammonium hydroxide was used as an additive.

Untargeted analysis of samples by DIMS allowed us to detect 3047 mass peaks in the three group samples. Only peaks that were common for all samples in each group were admitted to the analysis. Venn diagram construction enabled the visualization of the mass peak distribution between three group samples, which facilitated the further interpretation of the results (Figure 2). For all sample groups, 1985 mass peaks were common; 422 mass peaks were common for samples where n-butylamine and ammonium hydroxide were used; 26 mass peaks were common for samples where n-butylamine and formic acid were used; 34 mass peaks were common for samples where ammonium hydroxide and formic acid were used; and 327, 186, and 67 mass peaks were unique for samples where n-butylamine, ammonium hydroxide, and formic acid were used, respectively.

These results demonstrate the importance of the choice of additive for improving metabolite coverage. The use of n-butylamine showed the greatest superiority in global coverage of sample metabolomic composition in ESI(−) compared with other selected additives. The least amount of metabolomic coverage was observed with the use of formic acid. There were no interindividual variations in metabolomic composition between samples, as the same deproteinized pooled human plasma was used in all experiments. Thus, the ability of the additives to affect the ionization efficiency of the analyte was the most obvious cause of the observed differences in the numbers of detected mass peaks between the three group samples.

**Figure 3:**
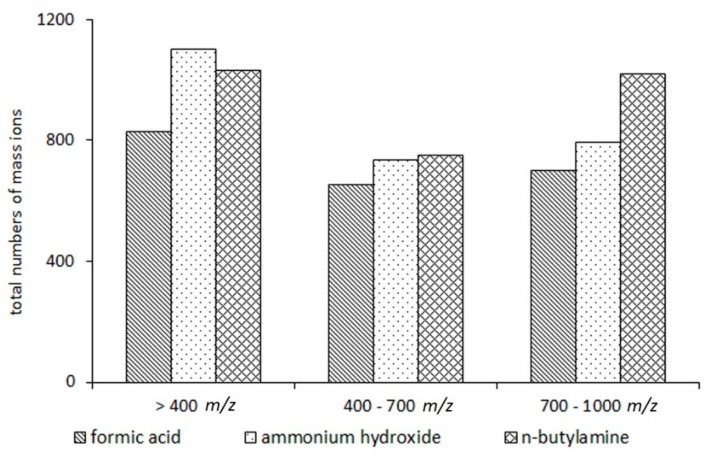
Comparison of the effect of the studied additives on the distribution of mass peak abundance across three segments of the MS spectrum in negative mode.

Despite the fact that among the three additives used in the study, the application of n-butylamine provided the most comprehensive metabolite coverage, a detailed comparative analysis of mass peak lists revealed the heterogeneity of their effect at various *m*/*z* (mass-to-charge ratio) ranges of the MS spectrum (Figure 3). For example, the greatest number of mass peaks in the lower *m*/*z* range of the MS spectrum (up to 400 *m*/*z*) was registered in the spectra of samples where ammonium hydroxide was used. In this range, amino acids, fatty acids, energy metabolism molecules, and so forth, are presented [29,30]. The greatest number of mass peaks in the higher *m/z* range of the MS spectrum (700–1000 *m*/*z*) was registered in the spectra of samples where n-butylamine was used. Phospholipids are detected in this range [30]. There was no significant difference in the number of mass peaks in the “middle” *m*/*z* range of the MS spectrum (over the range 400–600 *m*/*z*) between samples with n-butylamine and ammonium hydroxide. Compared with the mass spectra of samples with n-butylamine and ammonium hydroxide, the number of mass peaks in the mass spectra of samples with formic acid was smaller throughout all spectra.

The heterogeneity of the feature distribution throughout the regions of the MS spectrum is presented. The mass peaks were detected by direct mass spectrometry profiling in negative mode in plasma samples where the studied additives (formic acid, ammonium hydroxide, and n-butylamine) were used.

In the next stage of the experiment, the relative intensity of several common mass peaks from normalized spectra was compared. The mass peaks (with a clear isotope pattern) detected in various segments of the MS spectrum were randomly selected for the analysis: two mass peaks were taken from the range 80–200 *m*/*z*, two mass peaks were taken from the range 200–300 *m*/*z*, two mass peaks were taken from the range 300–400 *m*/*z*, and so forth. Putative annotation of the selected mass peaks was performed (Table 1).

**Figure 4:**
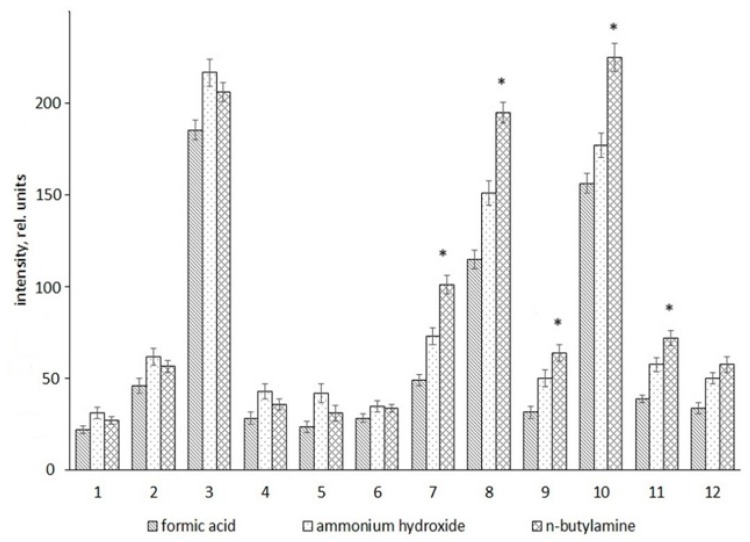
Comparison of the effect of the studied additives on the signal intensity of selected mass peaks. A comparison of the signal intensities of the selected mass peaks in the blood plasma samples where the studied additives (formic acid, ammonium hydroxide, and n-butylamine) were used is presented. Mean values (±SD) of signal intensities of mass peaks, which are differences between the compared groups, are shown in the figure. The signal intensity was normalized by means of an internal standard (* *p* < 0.05). Numbers correspond to the ordinal metabolite numbers indicated in Table 1.

Comparative analysis of MS spectra showed a difference in the ability of the studied additives to affect the signal intensity of mass peaks with various *m*/*z* values (Figure 4). For example, in the low-mass range of the MS spectrum (up to 400 *m*/*z*), the relative mass peak intensity in the spectra of samples with ammonium hydroxide was slightly higher than the intensity of the same mass peaks in the spectra of samples with n-butylamine. In contrast, in a higher *m*/*z* range of the MS spectrum (range 600–1000 *m*/*z*), the relative mass peak intensity in the spectra of samples with n-butylamine was significantly higher than the intensity of the same mass peaks in the spectra of samples with ammonium hydroxide. There was no significant difference in the relative intensity of mass peaks related to the middle *m*/*z* range of the MS spectrum (over the range 400–600 *m*/*z*) between the compared sample groups (data not shown). Compared with the mass spectra of samples with n-butylamine and ammonium hydroxide, the relative mass peak intensity in the mass spectra of samples with formic acid was smaller throughout all regions of the spectrum.

Thus, the results of the comparative analysis showed that n-butylamine is one of most effective additives for analysis of metabolite composition in ESI(−). It should be noted that the application of n-butylamine as an additive provided a significant increase in the number of detected mass peaks and an enhancement of signal intensity in the 700–1000 *m*/*z* range (phospholipids, triacylglycerols, etc.) compared with the traditionally used ammonium hydroxide and formic acid. Most likely, the results indicate the better ionization of high-molecular-weight metabolites.

**Figure 5:**
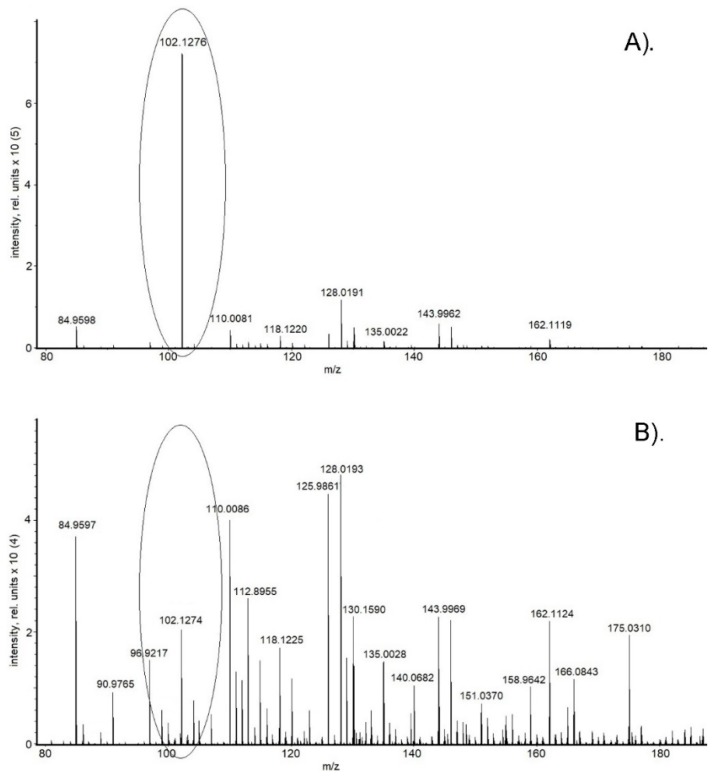
MS spectrum of methanol solution in ESI(+) after infusion of *N,N,N*-triethylamine (TEA)-containing mobile phases following flushing with a high volume of cleaning solution (50% aqueous acetonitrile by a syringe pump for 20 min, flow rate of 180 µL/h) (**A**) and following application of the proposed decontamination procedure (methanol, water, and isopropanol in equal proportions with 0.3% formic acid) (**B**). The peak corresponding to TEA (102.1277 *m*/*z* for TEA [M + H]^+^) is marked. The small range of the MS spectrum (80–180 *m*/*z*) is presented for clarity.

However, the ability of alkylamines to pollute the MS source is a barrier to their widespread use in metabolomics [22,23]. That is why special attention in the experiment was paid to controlling the purification procedure. An ordinary wash procedure (direct infusion of 50% aqueous acetonitrile by a syringe pump for 20 min) was run after each analysis. The effectiveness of the ordinary wash procedure was evaluated in both polarities: negative and positive. There were no traces associated with the use of ammonium hydroxide or formic acid after the ordinary wash procedure. At the same time, traces of n-butylamine were detected in the mass spectra after the ordinary wash procedure in positive mode. Flushing with a high volume of cleaning solution (50% aqueous acetonitrile) for a long time helped eliminate the contamination. As a result, the run time of the analysis (including the wash procedure) was significantly increased compared with the analysis where formic acid or ammonium hydroxide was used. Mixtures of various organic solvents in different proportions were tested to reduce the flushing time. After several failed experiments, the most effective decontamination procedure was found. The use of the solution (methanol, water, and isopropanol in equal proportions with 0.3% formic acid) and an increase in the gas temperature from 180 to 300 °C accelerated the process of n-butylamine trace removal. The average flushing time was 30 min (flow rate of 180 µL/h). About 10 min was needed as well for reducing the temperature to the initial value (180 °C) and its stabilization. We assumed that the proposed decontamination procedure would also be effective for the elimination other alkylamines, for instance, *N,N,N*-triethylamine (TEA). After using TEA as an additive for MS analysis and the ordinary washing procedure (direct infusion of 50% aqueous acetonitrile by a syringe pump for 20 min, flow rate of 180 µL/h), the polarity was switched from negative to positive and a highly intense peak corresponding to TEA (102.1277 *m*/*z* for TEA [M + H]^+^) [31]) was observed in the positive ion mass spectra (Figure 5A). There was no significant alteration in signal intensity after flushing with a high volume of ordinary cleaning solution (direct infusion of 50% aqueous acetonitrile by syringe pump) for a long time. The prolonged application of the proposed decontamination procedure (methanol, water, and isopropanol in equal proportions with 0.3% formic acid) allowed for a significant decrease of peak intensity (Figure 5B).

Unfortunately, the application of the proposed decontamination procedure did not completely eliminate the background of TEA. Probably, a significant part of TEA was eliminated, but some of it was adsorbed on the vacuum collector, quadrupole parts, and so forth. Regardless, the peak intensity (102.1277 *m*/*z*) was significantly reduced and did not exceed that which is constantly present in biological sample contaminant peaks, the elimination of which is very difficult (organic solvent clusters, phthalates, etc.). Minimization of ion suppression provided the ability to perform highly sensitive studies in ESI(+).

## 3. Discussion

The current trend in medicine is a shift from expensive, time-consuming, and painful patient diagnostic procedures to the application of noninvasive or minimally invasive approaches [28]. Metabolomics-based approaches represent one way of achieving this. Successfully using metabolic profiling for the discovery and identification of biomarkers that can predict the risk of disease progression and define the early stages of diseases has been demonstrated in numerous studies [32,33,34]. For example, the use of plasma and serum metabolic signatures provided improved risk prediction of lung cancer, interstitial cystitis, prostate cancer, coronary heart disease, impaired glucose tolerance, tuberculosis, and so forth [25,30,35,36,37,38,39].

The low ionization efficiency of some substances or their low concentration may prevent their detection in samples. An additive present in a sample may improve the ionization efficiency of the analyzed molecules and thus aid in enhancing the detection limit of the method. It should be noted that the increase in the overall number of registered mass peaks in spectra does not mean a proportional increase in the number of detected metabolites. Many of those registered in spectra mass peaks are isotopic masses, various adducts of metabolites, results of metabolite fragmentation, and so forth, which do not possess any biological information [40]. However, the increase in the total number of detected mass peaks also enables an increase in the number of “captured” metabolites.

Unfortunately, there are many difficulties and uncertainties which can complicate metabolomic analysis. The inability to perform MS/MS analysis due to the low concentration of metabolites in a sample or their low ionization efficiency, lack of clear isotope distribution pattern in mass spectrum, and so forth, prevent the correct annotation of much metabolites. In addition, difficulties with the mapping of a large number of identified compounds to known metabolic pathways can obstruct the identification of their biological functions [41]. All of these factors limit the correct interpretation of metabolic data. Nonetheless, the discovery of the metabolite patterns associated with any disease or specific disease states, as well as obtaining all possible information about the metabolite ions (*m*/*z* values, relative peak intensities, evaluation of peak ratios, etc.), can facilitate the creation of predictive mathematical models [1,41]. Based on such models, the physiological state (i.e., disease) can be identified. The accumulation of diagnostic information from the maximum possible number of metabolites associated with a particular disease is the most essential factor providing accurate disease diagnostics by metabolomic signatures. This is why approaches that can increase the metabolite coverage are so important for laboratory diagnosis in order to improve the accuracy of disease prognosis, monitoring of individualized treatment, and so forth.

The use of additives is one of the most effective and simple methods of increasing metabolite coverage. Alkylamine additives are widely used in LC-MS analysis of high-molecular-weight compounds [16,17,18,19,20,21]. The protocol design for the detection of high-molecular-weight compounds allows for neglecting the suppression effect of alkylamines that have accumulated on MS devices. Unfortunately, such contamination does not allow for using the same instrument to investigate low-molecular-weight substances in ESI(+). The difficulty of eliminating alkylamine contamination has been described in many studies [42]. These are very complex and extended procedures that require disassembly and washing of all components (ion transfer, ion exchange optics, and vacuum collector) by a service engineer [22]. The proposed decontamination procedure can be performed directly by the researcher and does not require the assistance of a service engineer. The procedure does not completely eliminate the contamination of an MS device associated with the use of alkylamine-containing mobile phases, but it does minimize the signal intensity of the peak that provided the performance to analyze low-molecular-weight compounds in ESI(+).

## 4. Material and Methods

### 4.1. Blood Plasma Sample Preparation

Venous blood for metabolomic analysis was taken from healthy volunteers (25–30-year-old males) prior to a morning meal and was collected into EDTA spray-coated tubes (BD Vacutainer; Becton, Dickinson and Company, Franklin Lakes, NJ, USA). The study was approved by the local ethical authorities. Then, these tubes were centrifuged for 15 min at 1600× *g*. The supernatant was aliquoted, transferred to clean plastic Eppendorf tubes (Eppendorf AG, Hamburg, Germany), and stored at −80 °C. Plasma deproteinization was performed by mixing plasma, water (LiChrosolv; Merck, Darmstadt, Germany), and methanol (Fluka, Munich, Germany) in a 1:1:8 (*v*/*v*) ratio. After thorough vortex mixing, the tubes were incubated for 20 min. Then, the sample was centrifuged at 14,000× *g* (Centrifuge; Eppendorf AG, Hamburg, Germany) at room temperature for 15 min. The supernatant was transferred to clean plastic Eppendorf tubes [35], and the tubes were divided into several equal parts (three groups of five tubes each). Before mass spectrometry analysis, 50 volumes of solution of methanol/water (9:1, *v*/*v*) containing one of selected additives (0.1% (*v*/*v*) formic acid (Fluka, Munich, Germany); 0.001% (*v*/*v*) ammonium hydroxide (Sigma–Aldrich St. Louis, MO, USA), or 0.0005% (*v*/*v*) n-butylamine (Sigma–Aldrich St. Louis, MO, USA)) was added to each tube. The resultant solution was subjected to mass spectrometry analysis.

### 4.2. Mass Spectrometry Analysis of Blood Plasma Metabolome

A hybrid quadrupole time-of-flight mass spectrometers (micrOTOFQ and maXis, Bruker Daltonics, Bremen, Germany) was set up for priority detection of ions with the *m*/*z* range from 80 to 1000. The samples were injected into the ESI source using a glass syringe (Hamilton Bonaduz AG, Bonaduz, Switzerland) connected to a syringe injection pump (KD Scientific, Holliston, MA, USA). The flow rate of samples to the ionization source was 180 µL/h. Spectra were recorded in negative ion mode.

### 4.3. Data Analysis

The MS raw data were processed using DataAnalysis software (version 3.4, Bruker Daltonics, Bremen, Germany) and converted into a peak list with the following parameters: peak width—4, S/N threshold—3, relative and absolute threshold intensity—0.01% and 70%, respectively. Peaks in different samples relate to the same metabolite ion if the mass difference between the peaks does not exceed 0.01 Da. Alignment of mass spectrum peaks and data correction to address ionic inconsistency in samples were performed using a self-made algorithm in Excel. All peak intensity and area values were normalized by the internal standard (IS) losartan (C_22_H_23_ClN_6_O, *m*/*z* = 421.1538) concentration levels [43].

Analysis of acquired data was carried out by means of PCA implemented in ProfileAnalysis (Bruker Daltonics, Germany). A Kruskal–Wallis *H* test was used to evaluate the statistically significant differences between compared groups.

### 4.4. Mass Spectra Peak Annotation

The identification was performed based on the use of two characteristics: accurate mass and isotopic abundance distribution. The search of annotated metabolites that had the closest *m*/*z* values to those obtained during the MS analyses was carried out by using reference databases: The Human Metabolome Database (HMDB) (http://www.hmbd.ca), METLIN (http://metlin.scripps.edu), and LIPID MAPS (www.lipidmaps.org). The mass tolerance window was 0.03 Da. Theoretical isotope patterns for each of the metabolites were produced using the Isotope Pattern Calculator (Bruker Daltonics, Germany). Thus, the metabolites were identified to level 2 (putatively annotated compounds) of the Metabolomics Standards Initiative (MSI) requirements [44]. It should be noted that some masses were redundant (they had several candidates). The candidates had identical brutto formulas and, consequently, identical isotope distribution. This fact did not allow for properly differentiating them in this study.

## Figures and Tables

**Table 1:** Putatively identified selected metabolites.

No.	Metabolites	HMDB ID	*m*/*z* Value		Elemental Composition
			Measured (*m*/*z*)	Calculated (Da)	
*1*	succinic acid	HMDB0000254	117.0195	117.0182	C_4_H_6_O_4_
*2*	malic acid	HMDB0000744	133.0157	133.0131	C_4_H_6_O_5_
*3*	palmitic acid	HMDB0000220	255.2405	255.2318	C_16_H_32_O_2_
*4*	α-linolenic acid	HMDB0001388	277.2201	277.2162	C_18_H_30_O_2_
*5*	arachidonic acid	HMDB0001043	303.2324	303.2318	C_20_H_32_O_2_
*6*	docosahexaenoic acid	HMDB0002183	327.2323	327.2318	C_22_H_32_O_2_
*7*	PG *	n/a	735.5201	735.5170	C_39_H_77_O_10_P
*8*	PG/PA *	n/a	747.5201	747.5170	C_40_H_77_O_10_P
*9*	PS *	n/a	808.5143	808.5123	C_44_H_76_NO_10_P
*10*	PS *	n/a	830.5935	830.5905	C_45_H_86_NO_10_P
*11*	n/a	n/a	936.5211	n/a	n/a
*12*	n/a	n/a	938.5012	n/a	n/a

Metabolites were annotated by library search (Human Metabolome Database, HMDB) and accurate mass and isotopic abundance distribution; *m*/*z*—mass-to-charge ratio; n/a—not assigned; PG—phosphatidylglycerol; PS—phosphatidylserine; PA—phosphatidic acid. * redundant masses with several candidates.
